# Addresses and Papers by the Members of the Instructing Staff of the New York State Veterinary College, at Cornell University, for the Years of 1896–98

**Published:** 1899-06

**Authors:** 


Addresses and Papers by the Members of the Instructing Staff of the New York State Veterinary College, at Cornell University, for the years of 1896-98. Ithaca, N. Y.: Published by the University, 1898.

This collection of fifty-eight papers and addresses, representing nearly 500 pages of matter, has been selected from the published work of various members of the instructing staff of the New York State Veterinary College during the two years of its existence. The work is now presented to the members of the medical and veterinary profession, to scientific men, stock raisers and, indeed, to all who are interested in the propriety of our great commonwealth, and the advancement of science.

To enumerate the papers even would be impossible to say nothing of a cursory review of each one—they are all of high character—scientific and reflect the work done by the brilliant faculty of our state veterinary college.

Professor Law and his confréres are to be heartily congratulated on their efforts and on the splendid showing which this school has made in such a brief space of time. Inasmuch as this volume is published by the university, it is probable that some copies are for gratuitous distribution and may be obtained by writing to Professor James Law. W. C. K.

